# Detector alignment for X-ray crystallography using *Millepede-II*

**DOI:** 10.1107/S1600576726001287

**Published:** 2026-03-26

**Authors:** Thomas A. White

**Affiliations:** a Deutsches Elektronen-Synchrotron DESY Hamburg Germany; b Center for Data and Computing in Natural Science CDCS, Hamburg, Germany; Uppsala University, Sweden; The European Extreme Light Infrastructure, Czechia

**Keywords:** detector alignment, X-ray crystallography, *Millepede-II*, serial crystallography

## Abstract

I describe a method for accurately refining the geometrical parameters of segmented X-ray area detectors on the basis of serial crystallography data, using ‘Millepede’ – an algorithm created for a very similar problem in high-energy physics. The Millepede method for serial crystallography builds on the approach of Brewster *et al.* [*Acta Cryst.* (2018), D**74**, 877–894], in which the detector parameters are refined simultaneously with the parameters for each individual crystal. This accounts for the mutual dependency between the parameters and thereby avoids the bias and slow convergence problems that have afflicted older approaches in which the deviations between observed and calculated Bragg peak positions were taken directly as the updates for the detector panel positions. The Millepede method uses the special structure of the least-squares normal equations to reduce them to a much smaller form that can be solved very quickly, even compared with the sparse matrix methods used previously. This makes it practical to refine the detector geometry frequently and thereby maintain accurate calibration without specialized alignment campaigns. Tilts of detector panels out of the plane can be reliably refined, as can the overall distance of the detector in the beam direction. With a simulated test case, the new method produced panel shifts within 7% of the correct values with only one iteration, and produced almost exactly correct shifts after a second iteration. A simulated out-of-plane panel rotation was correctly determined to within 0.001°. Applied to experimental data from an X-ray free-electron laser, the method increased the indexable fraction of frames from 30% to 91% in a single iteration, and to 96% after two further iterations. Computing the geometry updates on the basis of 2060 crystals took only 0.819 s on desktop computing hardware, including the time taken to read the required data from disk. The scaling was found to be very close to linear for up to 100 980 sets of crystal parameters, which took only 78.2 s to process under the same conditions. The method has been applied as part of a real-time feedback system at a synchrotron radiation beamline, in which an out-of-plane detector tilt of 0.04° was detected and corrected. Possible further applications are also described here.

## Abstract 2

A computational method for refinement of multi-panel detector geometry has been adapted from high-energy physics to serial crystallography. The method is many times faster and much more accurate than previous approaches.

## 1. Introduction

Accurate and precise information about the position and orientation of the detector is crucial to the success of many types of X-ray diffraction experiment. This is particularly true for serial crystallography (SX) experiments, which are sensitive to detector misalignments of less than one pixel’s width. Small detector misalignments manifest themselves as a reduced success rate for crystal orientation determination (indexing), or as systematic differences between the calculated and observed locations of Bragg reflections, which in turn lead to less accurate measurements of the reflection intensities. Large misalignments, of a size comparable to the separation between Bragg peaks, are likely to prevent indexing algorithms from succeeding at all, precluding further processing of the dataset. The improvements in scientific results produced by better detector geometry are well documented and can be substantial (Yefanov *et al.*, 2015 ▸; Ginn & Stuart, 2017 ▸; Brewster *et al.*, 2018 ▸).

**Figure 1:**
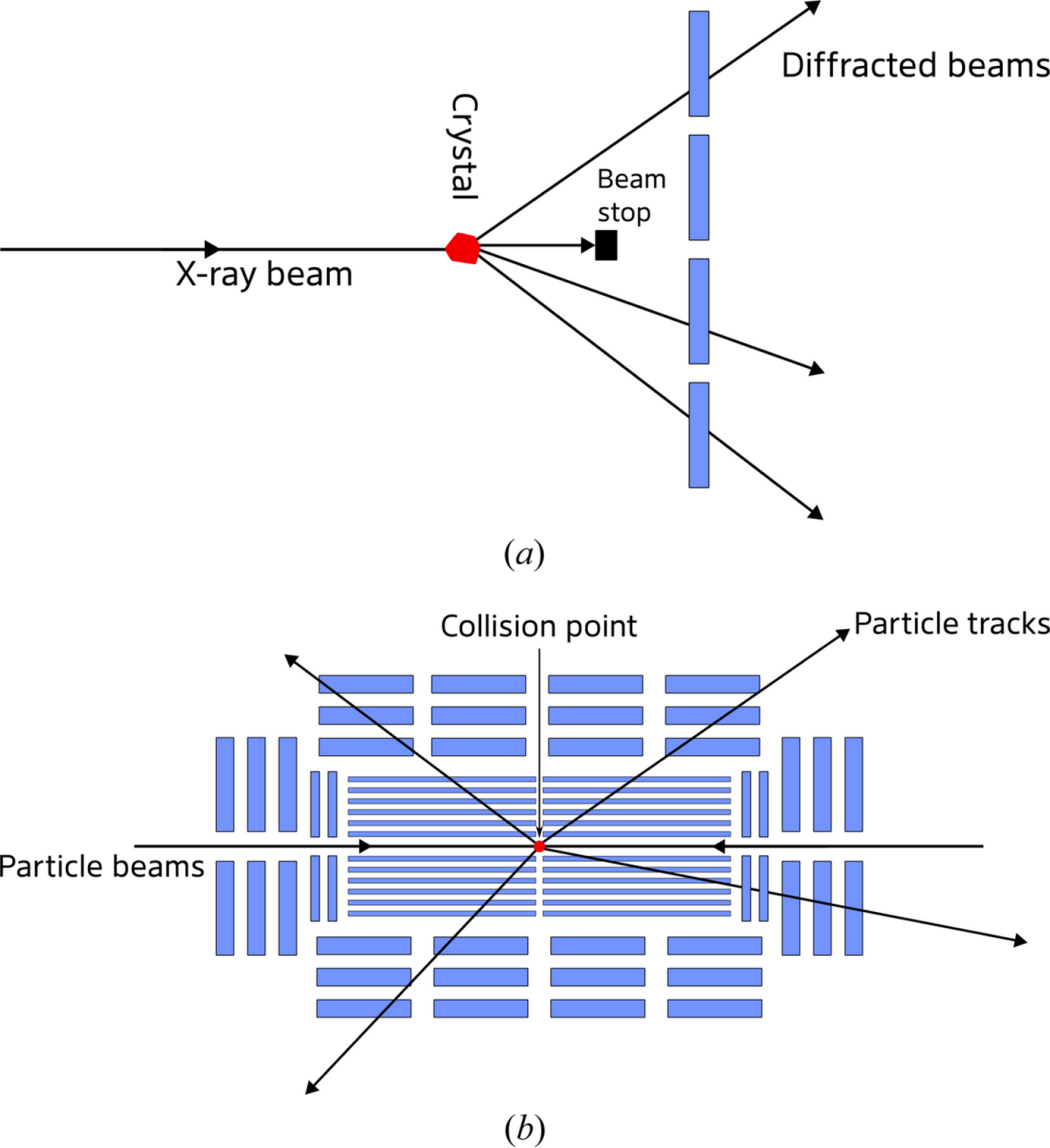
Schematic diagram comparison of (*a*) diffracted beams in a crystallography experiment with (*b*) particle tracks in a HEP experiment. Blue rectangles represent two-dimensional pixel detector panels oriented perpendicular to the plane of the diagram. In the case of the particle detector, the panels are arranged in a cylindrical pattern around the direction of the colliding particle beams.

The sensitivity to geometry of SX data arises because the images acquired consist of sharp well-separated Bragg peaks in regular geometrical patterns, each corresponding to the point where a diffracted X-ray beam intersects the detector [Fig. 1 ▸(*a*)]. These characteristics also mean that SX data can be used for refining the detector geometry. The most obvious method is to compare the observed positions of Bragg reflections with their calculated positions based on the result of the indexing procedure. If a systematic offset is found across many patterns, it seems likely that the detector panel is misplaced by that amount. This type of method, which will be referred to here as a ‘spot-deviation method’, is implemented in the program *geoptimiser* (Yefanov *et al.*, 2015 ▸) and has been used with reasonable success. Related methods improve on this by using extra information such as an intermediate result from the indexing algorithm (Ginn & Stuart, 2017 ▸), or by interleaving the process with refinement of diffraction parameters (Geng *et al.*, 2018 ▸).

A directly analogous detector-alignment problem exists in high-energy physics (HEP), where particle tracks intersect multiple layers of two-dimensional pixel detector panels arranged in cylindrical shells around the collision point, forming a three-dimensional detector [Fig. 1 ▸(*b*)]. The positions and orientations of the panels must be precisely known in order to achieve the scientific goals of the experiment (Kello, 2025 ▸). Each time a particle track intersects a detector panel, a peak is recorded that is similar in appearance to the Bragg peaks in crystallography. The tracks are usually helices because a magnetic field is applied within the detector to reveal the momenta of the particles. The spot positions can be calculated via a model of the helical particle track, which is derived from analysis of the observed spot positions. Just as with SX, if a systematic offset is found across many tracks, the assumed position of the detector panel should be updated.

Amongst the various solutions for detector alignment that have been developed in HEP, spot-deviation methods are explicitly recommended against. The problem with these methods is that the expected positions of the signals on the detector are calculated using a biased model in which the fitted parameters can ‘absorb’ part of the spot-position deviation. The observed spot deviations are therefore too small, and the refinement converges slowly or not at all. This was demonstrated for HEP with a simple ‘toy’ example (Blobel, 2006 ▸), and is demonstrated here for SX in Section 2.1.

These problems can be addressed by treating the alignment problem as a least-squares regression task in which updates to the detector geometry parameters and the many sets of crystal parameters are all calculated simultaneously. This has already been implemented for SX (Brewster *et al.*, 2018 ▸). Methods of this type, which will be referred to here as ‘full-matrix methods’, are free of the bias and convergence problems that afflict spot-deviation methods. However, they involve solving a very large matrix equation. For *N*_cryst_ diffraction patterns with *P* parameters per crystal, and *N*_det_ detector parameters, the square matrix representing the normal equations for the least-squares fit will have *N*_det_ + *PN*_cryst_ rows and columns. With a typical SX dataset consisting of many tens of thousands of crystals, the total number of parameters is very large and only a fraction of the data can be used before the refinement becomes computationally infeasible. The problem is somewhat alleviated by the use of sparse matrix methods in which only the non-zero matrix components are considered, but solving the normal equations still takes a large amount of computation. In HEP, this problem is particularly dramatic because of the much larger number of tracks and detector parameters: a HEP dataset may easily consist of millions of tracks and tens of thousands of detector parameters.

To address this, the ‘Millepede principle’ arose around 1996 (Blobel & Kleinwort, 2002 ▸; Blobel, 2006 ▸). The essence of this technique is to rearrange the least-squares normal equations to defer calculation of the updated track parameters, stopping once the updated detector parameters have been calculated. In HEP, while updated estimates for the individual track parameters may be useful, they are not used in practice because it would be better to re-analyze the entire dataset with improved geometry estimates to benefit from better data from the start of the process. Similar considerations apply to SX, where re-running the initial indexing step with improved geometry is likely to yield a higher number of successfully indexed diffraction patterns and more accurate assignments of indices to the observed spots. The Millepede method reduces the number of rows and columns in the matrix from *N*_det_ + *PN*_cryst_ to only *N*_det_, and this can be done without any approximations beyond the linear approximation inherent to least-squares fitting.

In this work, I show how the Millepede method for HEP can be adapted for use in SX. First, the problems inherent with the spot-deviation method are demonstrated by a simple simulation model (Section 2.1). The principles of least-squares methods for detector geometry refinement are then described (Sections 2.2 and 2.3) before extending them using the Millepede method (Section 2.4). Section 3 describes the implementation of the method into *CrystFEL* (White *et al.*, 2012 ▸), and this is followed by tests with simulated data (Section 4) and experimental data (Sections 5 and 6). In Section 7, the performance of the method is tested across a wide range of dataset sizes.

## 2. Methods

### 2.1. Bias with spot-deviation methods

The inherent bias of spot-deviation methods can be illustrated with a simple simulation. A short program was written, using the Julia programming language bindings to *CrystFEL* (version 0.12.0), to create ‘sketches’ of diffraction patterns and index them using a deliberately perturbed geometry with a known difference. The program performed the following steps:

(i) Choose a random crystal orientation.

(ii) Predict reflection locations on the basis of a random orientation and a model of the detector geometry.

(iii) Select half of the predicted reflections at random, to simulate that not all reflections would be found by a peak search.

(iv) Create a ‘Bragg peak’ data point for each selected predicted reflection. Round the peak locations to the nearest pixel, to simulate the positional uncertainty arising from a peak search.

(v) Attempt to index the diffraction pattern according to the peak locations and an artificially perturbed detector geometry model.

(vi) If indexing is successful, perform the usual refinement steps and record the offsets between the peak locations and their corresponding locations calculated using the indexing solution.

**Figure 2:**
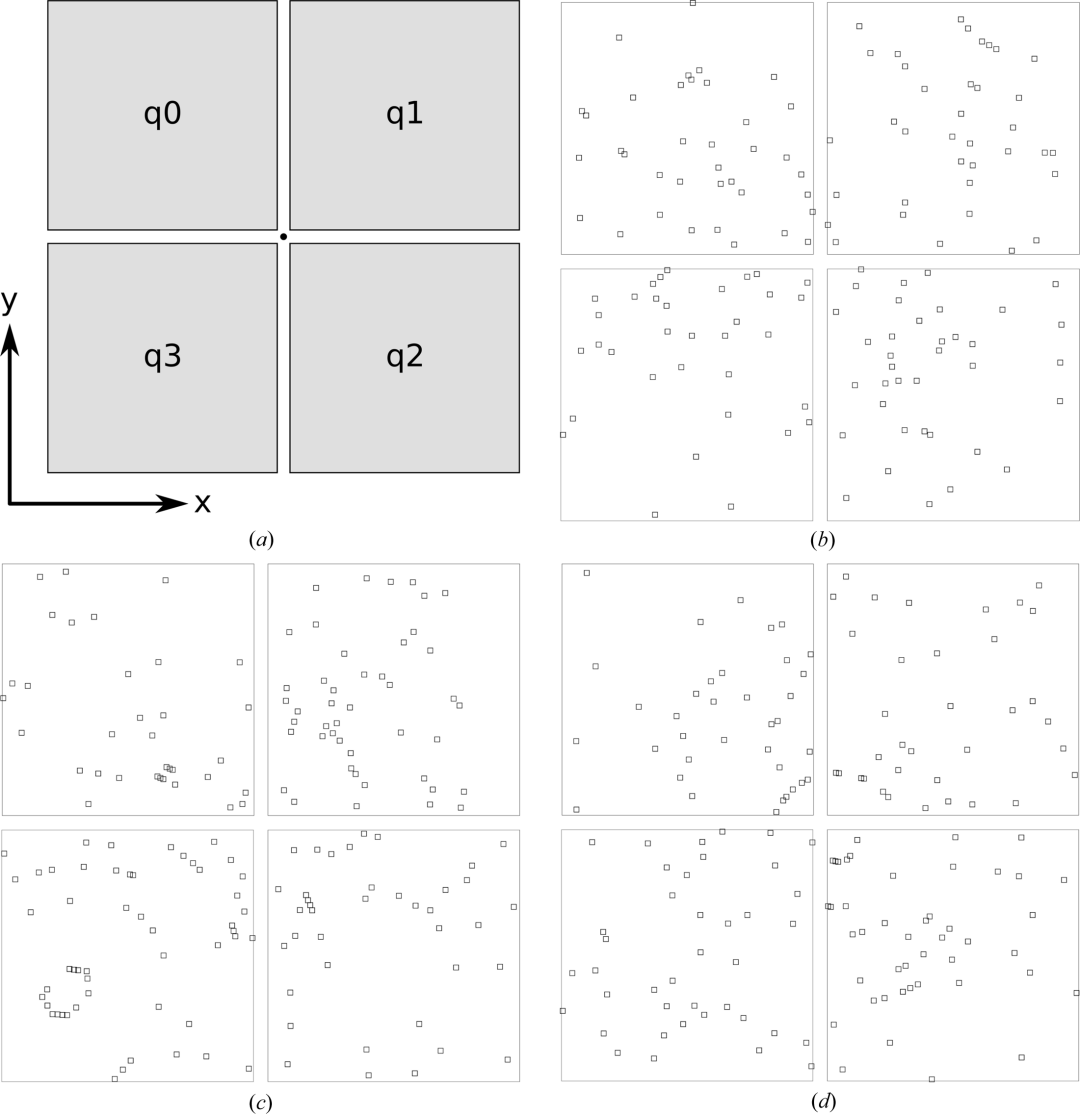
(*a*) Schematic diagram of detector panels in the simulated model, and the coordinate system used for the simulations. The positive *z* axis, which coincides with the X-ray beam direction, completes a right-handed coordinate system. The beam position is marked with a black dot. (*b*)–(*d*) Three representative simulated diffraction patterns, with Bragg peak locations marked by small squares.

The above process was repeated 100 times for a detector model with four panels, named q0, q1, q2 and q3, placed around the beam-center location, where each panel had a size of 1024 × 1024 pixels [Fig. 2 ▸(*a*)]. The simulated camera length was 100 mm, the pixels were square with width 100 µm and the X-ray energy was 9 keV per photon (1.3776 Å wavelength). The simulation was performed with a monoclinic primitive lattice, with *a* = 123 Å, *b* = 45 Å, *c* = 80 Å and β = 97°, and the average number of simulated peaks per pattern was 162. A selection of diffraction patterns are shown in Fig. 2 ▸(*b*)–2 ▸(*d*).

**Figure 3:**
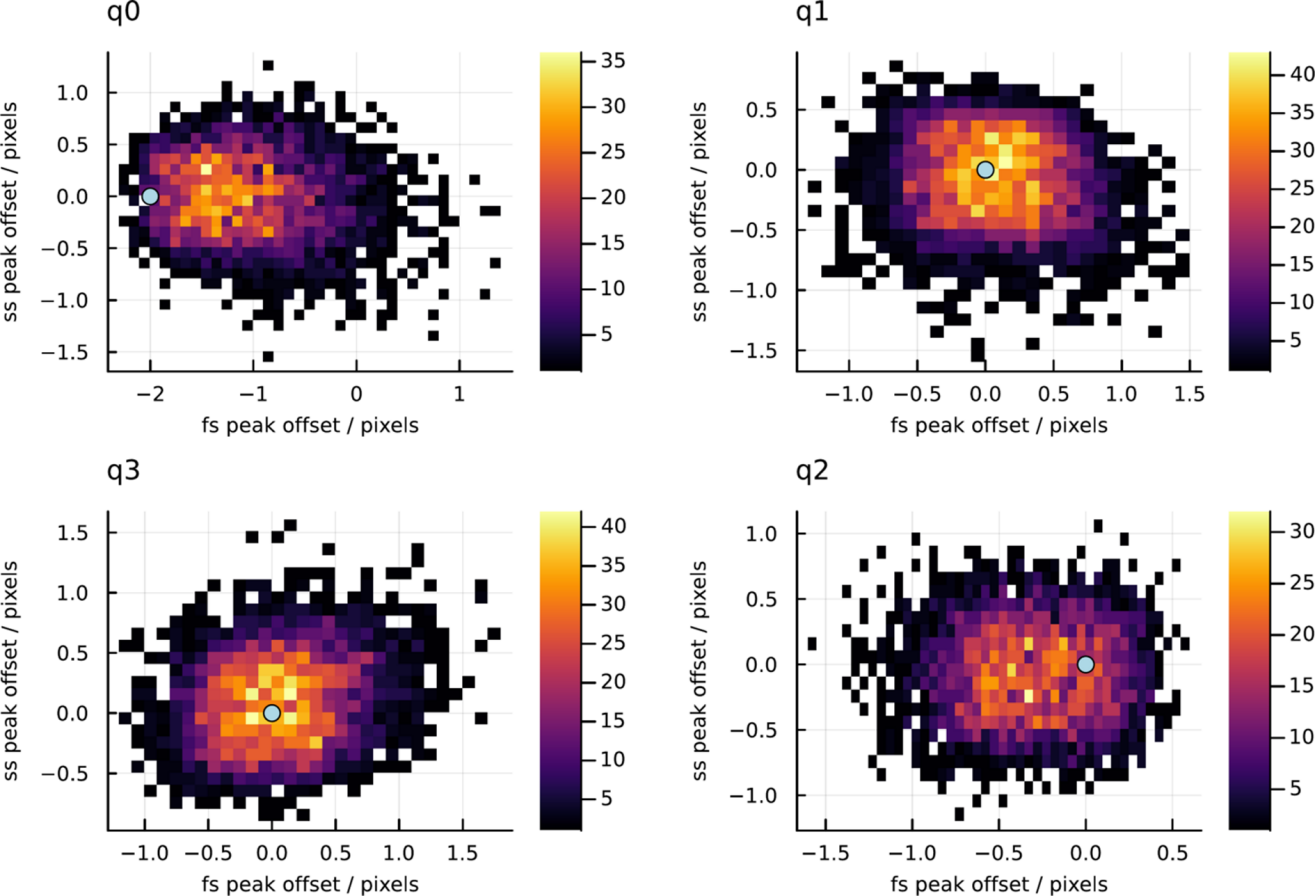
Two-dimensional histograms of spot-position offsets (calculated position minus observed position) from the simulation. The injected panel offsets are indicated by light blue dots.

Using the asdf indexing algorithm (White *et al.*, 2025 ▸) with retries and multi-lattice indexing both deactivated, all of the simulated diffraction patterns could be indexed. Fig. 3 ▸ shows two-dimensional histogram plots of the spot-position residuals in the fast-scan and slow-readout directions, which in this case (since there was no panel rotation) coincide with the *x* and *y* axes of the laboratory coordinate system, respectively. The injected detector offset, which is zero for all panels except q0, is marked with a light blue dot in each plot. The residual is defined as the calculated spot coordinate minus the observed spot coordinate, so the expected offset is negative when the geometry is perturbed by moving the panel in the positive *x* direction.

The distribution for q0 is offset from the origin in the correct direction, but by approximately half of the correct distance: the mean spot-position deviation was −106, −1.62 µm, compared with the correct injected value of −200, 0.00 µm. Panel q2, which was located across the beam center from the perturbed panel, shows a mean spot offset of −30.1, −7.04 µm, whereas the injected value was zero in both directions. Refinement using a spot-deviation method would, in this example, add a spurious movement to q2 while moving q0 by too little, producing an incorrect geometry.

### 2.2. Detector geometry refinement by least-squares fitting

The problems described in the previous section can be avoided by refining the detector parameters and crystal parameters in a single combined refinement, as was previously described for SX by Brewster *et al.* (2018 ▸). This section describes the implementation of this refinement within *CrystFEL*.

For each crystal *k*, the process begins by assigning Miller indices to observed spots on the detector using the pairing procedure described previously (White, Mariani *et al.*, 2016 ▸). For each pair, three quantities 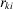 are calculated and stored, so the subscript *i* in 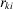 runs to three times the number of pairs found for crystal *k*. The first two quantities are the modeled coordinates minus the observed coordinates, in the fast-scan and slow-scan pixel directions, of the peak within the image data array for the detector panel that contains it. The third quantity is the modeled distance of the reflection away from the exact Bragg condition, under an assumption of a thin Ewald sphere. This distance will be referred to as ‘excitation error’, borrowing a term from electron microscopy. The excitation error of a reflection cannot be measured directly from a single diffraction pattern, but it is reasonable to assume that a visible reflection in a diffraction pattern is close to the Bragg condition, and helpful to include this information in the refinement by driving its value towards zero (Sauter *et al.*, 2014 ▸; White, Mariani *et al.*, 2016 ▸). To bring the real-space and reciprocal-space quantities to approximately the same scale and make them contribute approximately equally to the refinement, the excitation errors in m^−1^ are multiplied by 10^−7^.

In adapting methods from HEP to crystallography, it may seem that a ‘track’ in HEP should correspond to an individual Bragg reflection, because the diffracted beam emerging from the crystal is indeed a track, in a physical sense. However, for the purposes of data analysis, a better analogy for a particle track is an entire diffraction pattern, where one set of parameters (lattice parameters and orientation of the crystal) define all the spot positions. Let 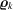 be a vector containing the parameters for diffraction snapshot *k*. This includes the crystal orientation as well as the lattice parameters and any related parameters subject to refinement. These will be referred to as the ‘local’ parameters. Let 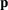 be a vector containing the detector-alignment parameters to be refined, which are common to all diffraction patterns and will be referred to as the ‘global’ parameters. The variation of 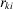 with changes to the local and global parameters will be modeled by 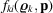, keeping the local and global parameters separate. For small adjustments to the parameters, the change in this function can be approximated linearly as
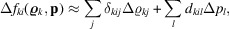
where 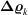 and 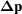 are the adjustments to be applied to the local and global parameters, respectively. The terms 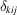 and 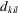 are the gradients of 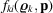 with respect to the local and global parameters. The subscripts *j* and *l* are indices over the local and global parameters, respectively: 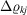 is the *j*th component of vector 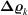 and 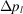 is the *l*th component of 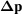. The linear approximation stated by the above equation is the only approximation made when using this method and is a reasonable assumption because the changes to the parameters should be small.

We would like to change the parameter vectors 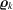 and 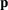 such that 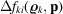 matches 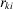 as closely as possible. Since an exact fit is unlikely to be possible, we instead minimize the weighted sum of squares of the differences between 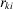 and 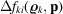,
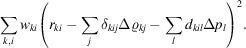
Taking the derivatives of this expression with respect to each of the parameters (local and global), and setting each one to zero, leads to the normal equations which can be solved for the parameter offsets under the linear approximation. For each global parameter, index *u*, there is a normal equation of the form
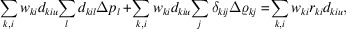
and for each local parameter, index *t*, an equation of the form
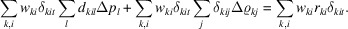
The combined set of normal equations can be written together in block matrix form as
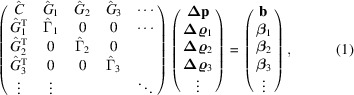
where the hat symbol denotes a matrix. The sub-matrices and sub-vectors have the following forms:
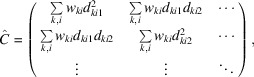

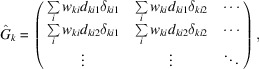

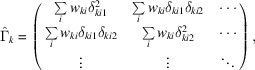

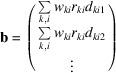
and
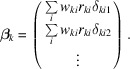
Sub-matrix 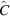 concerns only the global parameters, and each sub-matrix 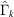 concerns only the local parameters for crystal *k*. The information about the dependencies between the local and global parameters is encoded by each sub-matrix 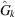. The sets of local parameters for each crystal do not directly affect one another – only indirectly via the global parameters – giving rise to the special structure of the matrix where all of the non-zero values are found in the top and left bands and the block diagonal.

### 2.3. The local fit

Neglecting the global parameters and considering only one crystal (index *k*) gives a reduced set of normal equations, which is referred to as the ‘local fit’, or in HEP as the ‘track fit’:
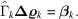
Solving this equation updates the local parameters for a single crystal, holding the global parameters constant. Such a refinement is performed by all SX data-processing software and in *CrystFEL* is called the ‘prediction refinement’ (White, Mariani *et al.*, 2016 ▸). Most implementations work in the way described above, including spot-position terms alongside the excitation-error terms in the refinement target function (Sauter *et al.*, 2014 ▸; White, Mariani *et al.*, 2016 ▸; Kabsch, 2014 ▸); however, one previous implementation used only the excitation-error terms and was still found to be beneficial to the final data quality (Ginn, Messerschmidt *et al.*, 2015 ▸).

In versions of *CrystFEL* before 0.11.0, the coordinate offsets of the beam center were also included in the local fit, in effect making a local estimate of the global beam center for each diffraction snapshot. The mean of the local estimates was then taken as the update to be applied for all patterns. This was removed in version 0.11.0 because, as noted by Brewster *et al.* (2018 ▸), this approach results in artefacts such as broadened distributions of the unit-cell parameters. Anecdotally, the mean of the local beam shifts was also found not to be accurate: even when a clear clustering of the values was seen, moving the beam by this amount did not always reduce the shift to zero in subsequent processing runs. This is another manifestation of the bias introduced by failing to consider the inter-relationships between the local and global parameters.

Although the eventual aim is to perform the global fit, a preliminary local fit for each crystal is still beneficial because it improves the crystal parameters regardless of whether a global refinement is carried out later. For a reason that will be described later, convergence of the local fit is a prerequisite for performing a Millepede global refinement.

### 2.4. The Millepede principle

Full-matrix methods work by solving equation (1) directly (Brewster *et al.*, 2018 ▸), yielding updated values for the entire set of local parameters as well as the global parameters. However, the number of rows and columns of the matrix is essentially proportional to the number of crystals in the entire dataset, which can easily be tens of thousands. The Millepede principle can be understood succinctly as the division of parameters into local and global sets as described above, followed by repeated use of a Schur complement (explained below) to eliminate the local parameters from the refinement. This results in a dramatically smaller matrix calculation with only as many rows and columns as there are global parameters.

Consider again equation (1), but with only one crystal, 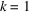:
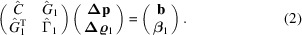
The set of linear equations represented by this matrix equation could alternatively be written as a set of two smaller simultaneous matrix equations:
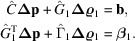
The term 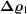 can be eliminated from the equations by first rearranging the second equation for 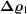, 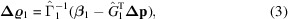
substituting into the first equation to get
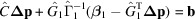
and rearranging to give
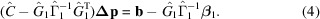
If 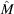 is the matrix on the left-hand side of equation (2), then the matrix 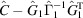 is known as the Schur complement of 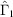 in 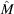. Solving equation (4) instead of equation (2) yields values for the global-parameter shifts, and takes into account the dependencies between local and global parameters, but involves a matrix where the number of rows and columns is reduced by the number of local parameters. No additional approximations have been made to do this, but the price paid for the size reduction is that it does not yield shifts for the local parameters.

Now consider a fit for two crystals, again including the global parameters, after reducing the matrix size for the first crystal as described above. The normal equations can easily be combined:
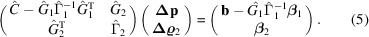
Applying the same matrix-reduction technique to this equation produces
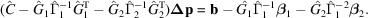
Since the correction terms for each successive set of local parameters are simply added together, the result for all crystals combined is given by
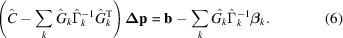
No matter how many crystals are considered, the size of the matrix equation does not change. Furthermore, provided that the local fit for each crystal has converged, the correction on the right-hand side of the equation (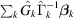) is equal to zero and can be neglected. This is, however, not always the case for HEP data analysis and is not assumed by the software.

## 3. Implementation of Millepede within *CrystFEL*

The Millepede algorithm for HEP is implemented in a piece of software, also called *Millepede*, written in Fortran. An interface to this program has been implemented within the *CrystFEL* software suite (White *et al.*, 2012 ▸) to adapt it for SX experiments. Originally, *Millepede* was a single program that performed track fitting and detector alignment together. In 2003, with the release of the second major version of the software (*Millepede-II*), the program was split into two software components, *Mille* and *pede*. *Mille* is a small routine that writes out data files containing the quantities 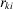, their errors 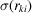, and their local and global gradients 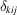 and 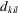. This routine is intended to be built into the software used for track fitting or other analysis. The data files can be read by the second program, *pede*, which performs the bulk of the refinement calculation by accumulating and solving equation (6) as well as doing rounds of outlier rejection and data validation. At the end of its run, *pede* outputs the correction values 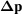.

Implementations of *Mille* are already available in C++ and Fortran, but for *CrystFEL* a new version was written in C from scratch, based on the documented file format and examination of the C++ version. This routine is called during the prediction-refinement stage of processing inside *indexamajig*, which is *CrystFEL*’s tool for indexing and integrating large numbers of diffraction snapshots. The *Mille* routine writes a new data file consisting of a series of records, one for each crystal lattice that could be successfully refined after indexing. Each record contains the relevant values for all peak–reflection pairs in one diffraction pattern.

Most of the required numbers are already needed by the prediction-refinement procedure, so the data needed for geo­metry refinement can be written with very little further calculation. Using the profiling tools previously described (White *et al.*, 2025 ▸; Gasparotto *et al.*, 2024 ▸), the mean time taken to calculate and write the calibration data was measured as 0.372 ms per frame, a negligible fraction of the mean overall processing time of 2250 ms per frame.^1^ The weighting factors for the measurements were all equal to 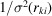, which is the default behavior of *pede*. The standard errors of all coordinate measurements were set to a constant value of 0.3 pixels, and the standard errors of all excitation-error measurements, after the scaling factor of 10^−7^ mentioned in Section 2.2, were set to 0.2.

A new program was written within the *CrystFEL* suite, called *align_detector*. This program constructs a file containing instructions for *pede*, runs *pede* itself, and then reads the parameter corrections and writes an updated *CrystFEL* geometry file based on the one used for the previous indexing run. The entire runtime for *align_detector* was found to be very short, below 10 s for typical dataset sizes (around 10 000 crystals), and is tested in Section 7. No provision was therefore required for sending the job to a high-performance computing cluster using the *CrystFEL* graphical user interface, as has been implemented for the indexing, merging and other steps.

The global parameters were defined as the translations and rotations of groups of detector panels at different hierarchy levels, as described in Section 3.1. The local parameters were defined as the free lattice parameters according to the lattice type of the crystal, and three rotation angles representing adjustments of the crystal orientation around the Cartesian axes *x*, *y* and *z*. There is no requirement for the same definitions of the local parameters to be used for every crystal, and therefore no requirement for each crystal to have the same (or even similar) lattice parameters.

Many options are available to control *pede*, including a choice of different strategies for solving the matrix equation. HEP detectors can consist of thousands of sub-detectors, so the global-parameter matrix is large and still has special structure, allowing the computation to benefit from additional strategies such as Cholesky decomposition or diagonalization. In this work, however, the number of global parameters is very small, certainly far below 1000, and so the basic strategy of matrix inversion can be used without difficulty.

### 3.1. Hierarchical refinement and constraints

**Figure 4:**
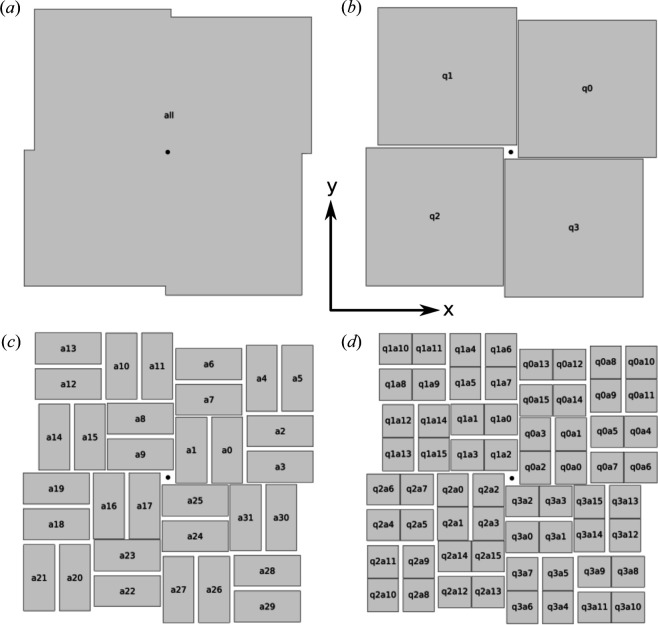
Schematic diagram of CSPAD represented at different levels of hierarchy: (*a*) level zero, as a single rigid unit; (*b*) level one, as four quadrants that can move independently of one another; (*c*) level two, as 32 silicon sensor chips; and (*d*) level three, as 64 readout chips. The coordinate system is shown at the center of the diagram, with the positive *z* axis, which is the beam direction, completing a right-handed coordinate system out of the plane. The approximate beam position is indicated by the black dot at the middle of each diagram.

For the purposes of alignment, a complex segmented detector is best described with a hierarchical structure. For example, the 1.5-megapixel Cornell–SLAC Pixel Array Detector (CSPAD) (Carini *et al.*, 2013 ▸) available at the Linac Coherent Light Source (LCLS) consists of four quadrants that can be moved relative to one another by motors [Fig. 4 ▸(*b*)]. Each quadrant is made up of eight silicon sensors [Fig. 4 ▸(*c*)], each backed by two separate readout chips [Fig. 4 ▸(*d*)]. The size of each readout chip is 194 × 185 pixels, with square pixels of width 110 µm. Refinement of the detector can be performed at four levels: the entire detector, the quadrants, the silicon chips and the individual readout chips. Since the readout chips are physically bonded to the silicon chips, they cannot move relative to one another. Nevertheless, it is conceivable that their modeled positions might not be exactly correct and may still need refinement.

Hierarchical refinement requires additional constraints. Consider for example if one parameter were to be defined for the overall detector translation and four additional parameters for the translations of the detector quadrants in the same direction. The whole detector could be moved either with the top-level parameter or by changing all the quadrant parameters by the same amount. There will therefore be a degeneracy between the parameters and the refinement will fail due to a rank-deficient normal matrix. This can be avoided by eliminating one of the translation parameters or by constraining the translations of the quadrants to sum to zero. The latter approach was used in *CrystFEL*, taking advantage of a mechanism in *Millepede-II* to constrain the sum of any given set of parameters to zero. The translations in all three directions, as well as all rotations, were required to sum to zero at every level of the hierarchy, allowing the next level up in the hierarchy to control the overall motion.

The geometry of an SX experiment is insensitive to the overall rotation of the detector around the beam direction. Such a rotation would only change the crystal orientations determined by indexing, rotating them around the beam without changing the set of excited Bragg reflections. Accordingly, the overall rotation of the detector around the beam was constrained to zero. A similar phenomenon appears in post-refinement using crystallographic data (White, 2014 ▸; Rossmann *et al.*, 1979 ▸).

The rotation of each panel group was defined with the three rotation axes going through the center of the group along *x*, *y* and *z*. The center was defined as the mean of the center coordinates of the groups at the hierarchy level below, recursively downwards until the individual panels are reached. The centers of the individual panels were defined as the mean coordinates of their four corners. This means that the top-level rotation center does not necessarily coincide with the beam position. The beam and rotation centers will be close together if the detector panels are laid out symmetrically around the beam, but this is not always the case, *e.g.* for the Jungfrau 16M detector installed at the Alvra Prime station at SwissFEL (Nass *et al.*, 2020 ▸). Although this creates some potential for confusion, it is not a problem for the method, because a rotation around the beam center can be equivalently described as a rotation around any other point combined with a translation perpendicular to the rotation axis.

### 3.2. Weakly constrained modes

Certain combinations of parameter changes are weakly determined by the experimental setup, or perhaps not determined at all. The overall rotation of the detector around the X-ray beam, mentioned above, is one such combination. However, more complicated combinations may exist and need to be accounted for. These ‘weak modes’ are a well-known problem in HEP and are said to be the main limitation to refinement accuracy in HEP (Bilka, 2022 ▸).

In HEP, weak modes can be mitigated by including different types of data. For example, data from particle collisions, forming tracks that radiate out from the center of the detector, can be combined with data from cosmic rays that produce tracks passing vertically downwards through the detector volume (ALICE Collaboration, 2010 ▸). There is also the option of deactivating the magnetic field normally used to bend the particle tracks into helices and thereby reveal their momenta (CMS Collaboration, 2014 ▸). Unfortunately, no analogous methods are immediately obvious for crystallography experiments.

Compared with spot-deviation methods, and in common with full-matrix methods, the Millepede method is more sensitive to weakly constrained modes. Since it directly constructs and solves the normal equations, if the matrix system includes eigenvectors with very small eigenvalues, large shifts will be produced. This would appear to be a disadvantage of the method, but it could actually be considered as an advantage. Any problems related to inadequately constrained parametrization of the detector will affect all methods of geometry refinement, although slow convergence may hide their effect. With Millepede or a full-matrix approach, such problems will be immediately obvious and can be investigated and fixed. They can be isolated, amongst other ways, by examining the eigenvectors of the normal matrix, finding those with small eigenvalues and then applying additional constraints using the *pede* control file. Similar procedures are possible with all full-matrix methods because the required information is contained within the normal equations of the least-squares fitting procedure.

Fortunately, significant problems with weak modes were not experienced in this work. One exception arose when attempting to refine out-of-plane tilts of the entire detector with a triclinic unit cell, *i.e.* allowing all six crystal lattice parameters to vary. With the beam in the *z* direction, tilting the detector around the *x* axis appears similar to ‘squeezing’ the entire crystal along the *y* axis, and similarly with a rotation around the *y* axis and squeezing along the *x* axis. Without the inclusion of the excitation-error terms in the refinement target function, the resulting parameter degeneracy results in a rank-deficient normal matrix and complete failure of the refinement. Including the excitation-error terms mitigates the degeneracy because the compressed lattice parameters would move reflections away from the Bragg condition. This was found to already permit successful refinement of the detector geometry. The problem does not occur at all with any lattice types other than triclinic, even without the excitation-error terms, because the restrictions on the lattice parameters would be violated by the squeezing. This is demonstrated in the next section.

## 4. Test with simulated data

To test the alignment method, the simulation described in Section 2.1 was repeated with a more complicated perturbation to the detector geometry model. The perturbed geometry was constructed by moving one panel (q0) by 200 µm along the *x* axis, rotating another panel (q2) by 0.5° around a vector through the panel center along the *y* axis and moving the entire detector by 1 mm along the *z* axis (beam direction). *Mille* data were additionally collected during the indexing step but the procedure was otherwise as described in Section 2.1.

The new refinement tool was then run on the *Mille* data produced from the indexing. Each detector panel was allowed to translate and rotate independently in all three dimensions. As described in Section 3.1, the translations and rotations were constrained to sum to zero in each direction at each level of the hierarchy below the topmost level. Top-level translations and rotations of the entire detector were allowed, except for rotation around the beam direction. The total running time was measured as 174 ms, including the time taken to load the *Mille* data from disk, in a single thread on a desktop computer equipped with a four-core (eight thread) Intel Xeon W-2225 processor at 4.1 GHz clock frequency and 16 GB of memory.

The refinement produced the geometry corrections shown in Table 1 ▸, where the refined corrections are compared with those applied by the perturbation (labeled ‘injected’). Here, the translations are those experienced by the corners of the panels, so the out-of-plane rotation of panel q2 about its center has resulted in an additional translation of the corner by 0.447 mm in the *z* direction and a very small translation in the *x* direction. The corrections determined by the refinement procedure are given in the same way, which allows them to be directly compared regardless of the levels of detector hierarchy at which movements were applied. The corrections aim to cancel out the geometry errors and so have opposite signs to the injected shifts

After one iteration, the refined values were already within 7% of the correct values, with the largest error being for the translation of panel q0 (0.187 mm in contrast to a correct value of 0.200 mm). A second round of indexing and refinement was performed using the updated geometry, producing a new set of correction values that were much smaller. The corrections from the two runs can simply be added together to determine the overall corrections, which for practical purposes exactly match the correct values: all the rotations were within 0.002° of the correct values, and the largest translation error was 4 µm (1.443 mm in contrast to a correct value of 1.447 mm).

To test the contribution of the excitation-error terms, the entire test was repeated without using the excitation-error values. This produced almost the same results (shown in Table 2 ▸). No difficulties were encountered with a rank-deficient matrix in this case, demonstrating that the squeezing mode described in Section 3.2 has been sufficiently constrained.

## 5. Experimental verification

At the time of writing, multiple anecdotal reports have been received by the author about successful applications of the technique, even in the hands of non-expert users. One case is presented here as a demonstration, based on a dataset that has previously been used as a tutorial dataset for *CrystFEL* and that is known to contain large geometry errors.

A sample of 2104 frames was taken of dataset 21 from the Coherent X-ray Imaging Data Bank (CXIDB) (Maia, 2012 ▸). This dataset consists of diffraction patterns from the human serotonin receptor 5-HT_2B_, in complex with ergotamine, which was measured at the CXI endstation of the LCLS as an initial demonstration of measuring G-protein-coupled receptors in a lipid cubic phase delivery medium (Liu *et al.*, 2013 ▸). The patterns were recorded on the CSPAD, as depicted in Fig. 4 ▸. The dataset is described in more detail elsewhere (White, Barty *et al.*, 2016 ▸). Using *CrystFEL* version 0.12.0, the patterns were indexed with *xgandalf* (Gevorkov *et al.*, 2019 ▸) in fast mode (—*xgandalf-fast*) applied to the peak search results deposited with the images, which came from a previous hit-finding stage of data processing. Multi-lattice indexing was activated, and the unit-cell tolerances were 5% of the target axis lengths and 1.5° for the cell angles. The target unit cell was orthorhombic C with axis lengths 61.65, 122.94 and 168.89 Å.

**Figure 5:**
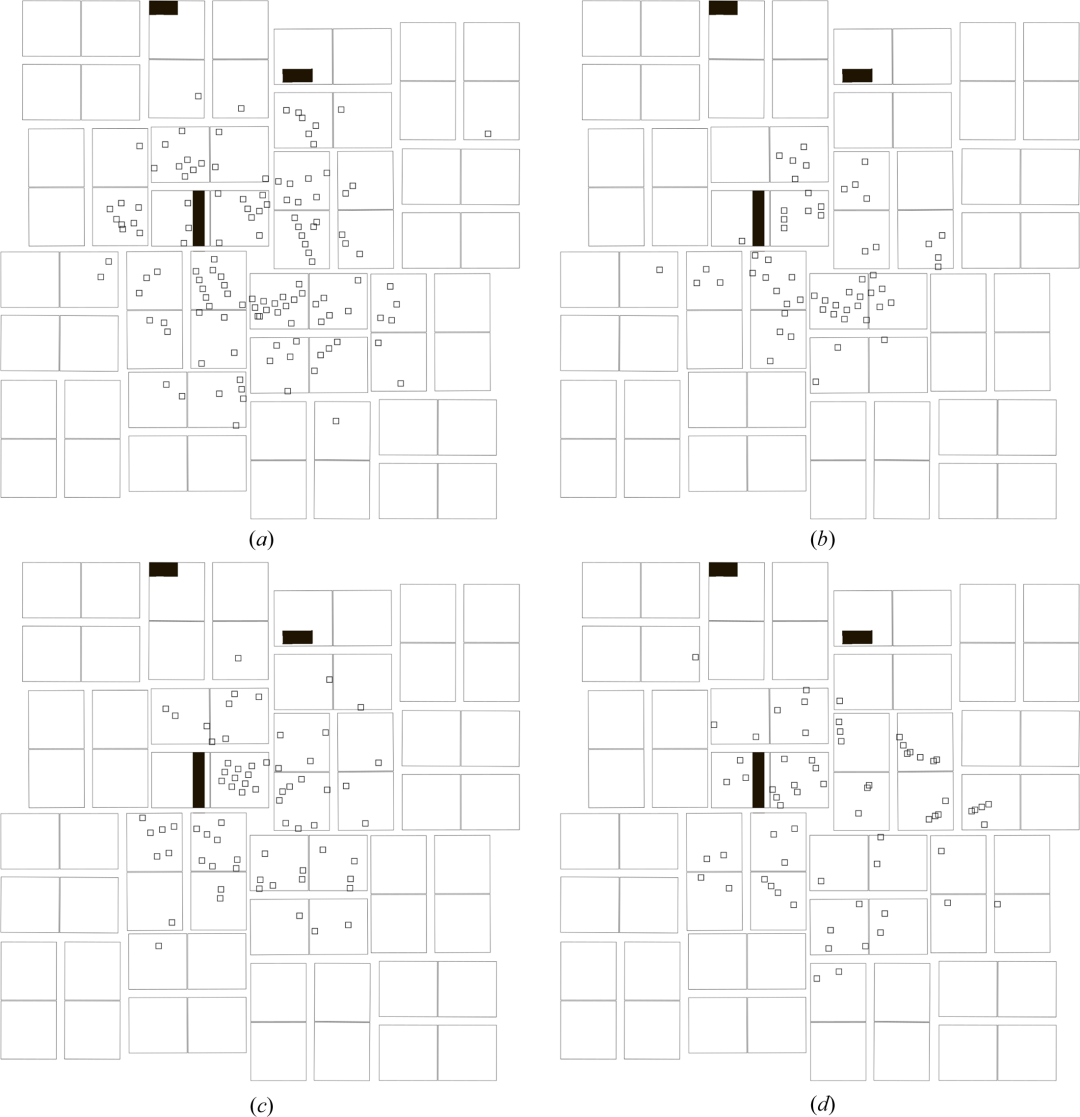
Geometrical views of representative diffraction patterns from human serotonin receptor 5-HT_2B_, in complex with ergotamine. The panel edges are shown corresponding to Fig. 4 ▸, and peak locations from the peak search are marked with small squares. Filled black regions were marked as untrusted due to defective pixels.

The detector hierarchy for the CSPAD was set up as described previously and shown in Fig. 4 ▸. Each of the 64 readout chips [Fig. 4 ▸(*d*)] has a name of the form q*X*a*Y*, where *X* is a number specifying which quadrant of the detector it belongs to and *Y* specifies the chip within the quadrant. Both sets of numbering start at zero. Each pair of numerically adjacent chips within one quadrant, for example q1a14 and q1a15, share a silicon sensor chip, as shown in Fig. 4 ▸(*d*). These names are used in the geometry files as well as in Tables 3 ▸ and 4 ▸. The peak locations from four representative diffraction patterns are shown in Fig. 5 ▸.

The first round of indexing was performed using the geometry file deposited at the CXIDB, with minor modifications to update the format to be compatible with recent *CrystFEL* versions and to add the hierarchy information. Overall, 635 frames could be indexed (30% of all frames), yielding results from 638 crystals (three frames contained two overlapping crystal diffraction patterns). On the basis of Millepede data recorded from the initial indexing run, the detector geometry was then refined using *align_detector* at the level of detector quadrants, restricted to in-plane motions only.

A second round of indexing was then performed using the refined geometry. This time, 1910 frames could be indexed, 91% of all frames and three times the original number, yielding results from 1947 crystals. A second round of geometry refinement was performed on the basis of these results, also at the level of detector quadrants but with out-of-plane translations, out-of-plane rotations and overall camera-length adjustments allowed. This refinement round detected and corrected a camera-length error of 2 mm. This was followed by a further indexing run in which 2012 frames were indexable (96%) with 2060 crystals found, and a further geometry-refinement run with the same settings as the second. The total panel movements, summed over all three refinement runs, are shown in Table 3 ▸. The largest translation of the corner of a panel in the beam direction, which includes the shift induced by out-of-plane rotations, was 2.9 mm for panel q0a13. In-plane shifts were found up to 0.786 mm or 7.15 pixels (panel q0a6) and there were in-plane rotations up to 0.13° (quadrant 0, panels q0a0 to q0a15). The largest out-of-plane rotation was 0.74° for quadrant 1 (panels q1a0 to q1a15). All of these corrections would be considered as problematically large for an SX experiment and demonstrate the ability of the new approach to correct significant geometry errors.

The time taken by *align_detector* was measured as 819 ms, on the third geometry-refinement round and including the time taken to read the *Mille* data from disk, under the same conditions as described in the previous section.

A fourth and final indexing run was then performed, in which 2010 frames were indexable with 2054 crystals found, practically the same numbers as the previous run and suggesting that the procedure had converged. To test the convergence, and also to check the stability of the refinement procedure, a fourth round of refinement was then performed. The panel movements from the fourth refinement run are shown in Table 4 ▸. They are very small compared with those in Table 3 ▸ except for the out-of-plane rotations within quadrant 3, which are small but not negligible at 0.3°. After a fifth indexing and refinement run, this rotation was close to zero (0.004°).

## 6. Incorporation within a real-time data-processing pipeline

Aside from convenience, the low computational requirements make the Millepede technique useful as part of an automated data-processing pipeline at an XFEL or synchrotron radiation beamline. The relative position of the detector can be checked automatically after every data-acquisition run, constantly monitoring the calibration and ensuring that any drifts are followed and changes are noticed. This fulfills one of the prerequisites for fully automated real-time processing: that the system must be fully calibrated before the data acquisition. A test system following this principle has been in use since 2023 for SX experiments at PETRA III beamline P11, monitoring the detector geometry after every run as part of the Amarcord database system described recently as part of our real-time data-processing system (White *et al.*, 2025 ▸).

**Figure 6:**
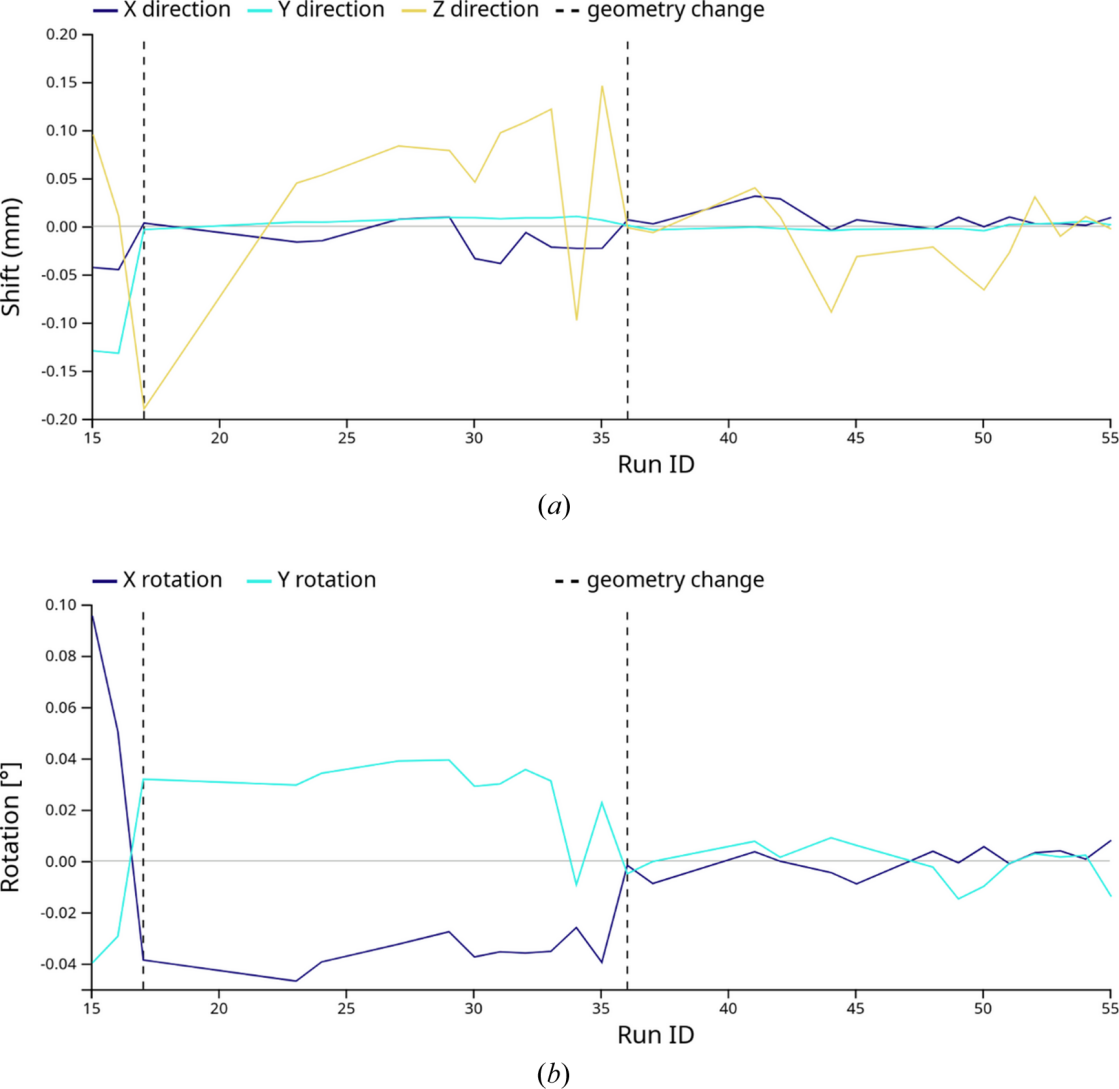
Graph of (*a*) translational and (*b*) rotational detector refinement parameters for the 16-megapixel detector at P11, during a two-day period of beam time. Vertical dashed lines mark times when the reference geometry was updated.

Fig. 6 ▸ shows the detector shifts for every data-acquisition run over a 48-hour period of beam time in June 2025. The detector was a Dectris EIGER2 X 16M, with a pixel width of 75 µm, consisting (from the software point of view) of a single rigid panel. The detector was mounted on a motorized tower that was moved by about 1 m away from the sample between runs, to allow access to the sample delivery system. Over this period, the reference geometry was updated twice, marked with vertical dashed lines. At the start of the experiment run, the detector-position estimate was found to be in error by about 50 µm in *x* and 120 µm in *y*, or 1.7 pixel widths, which was corrected by the first geometry update after a few test acquisitions. The beam-center coordinates in the plane of the detector were found to remain stable to less than one pixel (±50 µm) over the entire beam-time period. The detector position appears to be more stable in the vertical (*y*) direction than in the horizontal direction. Similar results have been observed in previous beam-time runs, and we do not yet know whether it is due to a mechanical property of the movable detector support tower, the focusing optics or another source. Larger variations are visible in the *z* direction, which may be due to the greater difficulty of measurement. Consistent values were determined for the small out-of-plane rotation angles (∼0.04° and 0.03° around the *x* and *y* axes, respectively) between runs 17 and 35, their influence being corrected by an update of the geometry at run 36.

## 7. Performance benchmark

**Figure 7:**
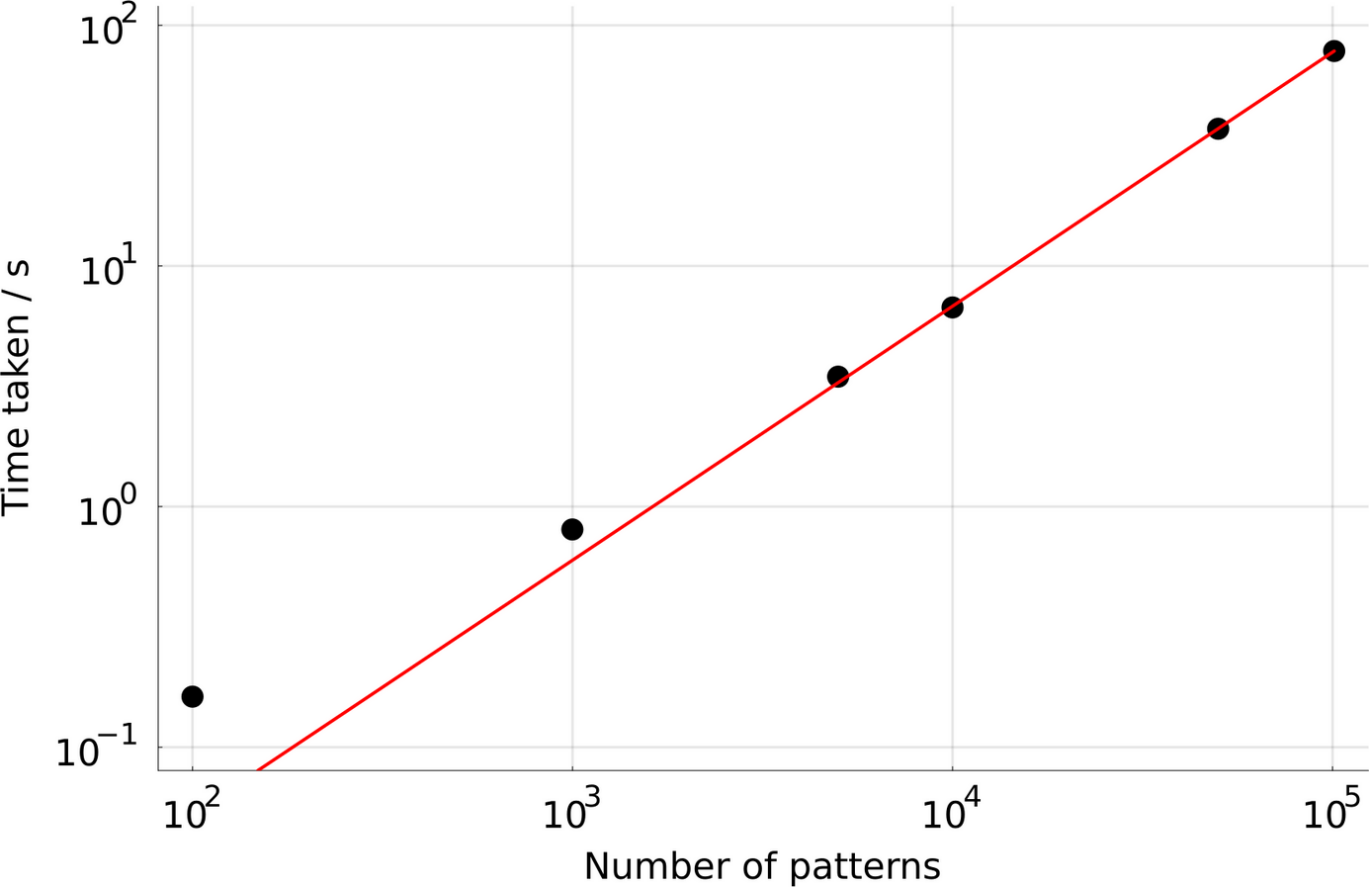
Time taken to refine a detector model as described in Section 4, for a wide range of dataset sizes. The red line is a power-law fit of the form 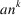, where *a* = 0.0004075 and *k* = 1.056.

To test how the performance scales across dataset sizes, the simulation from Section 4 was repeated, but with the number of patterns varying between 100 and just over 100 000. In all cases, the detector parameters refined to values close to those given in Table 1 ▸. For each number of patterns, *align_detector* was run four times and the mean of the last three runtimes taken. The results therefore include the time taken to load the data with a ‘warm’ disk cache. The mean runtimes are shown in Table 5 ▸ and plotted in Fig. 7 ▸. A power-law function of the form 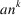 was fitted to the results, which showed that the scaling was very close to linear with *a* = 0.0004075 and *k* = 1.056. The times deviate from linearity for the lowest numbers of patterns, where the time was less than 1 s and overheads from other parts of the process become significant.

The scaling behavior, as well as the overall times, may be compared with those given previously (Brewster *et al.*, 2018 ▸) for the non-linear Levenberg–Marquardt algorithm using a sparse matrix routine in the Eigen library. There, the scaling exponent was larger (1.13) and the absolute times much longer: the largest run of only 5000 patterns took around 1000 s (16 min), around 280 times slower in absolute terms. To make an estimate of the time taken for one cycle of the non-linear least-squares method, this figure should be divided by ten. Allowing a further factor of two for differences in computer performance, the Millepede method is still around 14× faster. This figure even neglects the fact that *pede* performs rounds of outlier rejection, in effect solving the matrix equation 2–3 times per run.

The Millepede approach does not involve calculating updates for the crystal parameters, which would seem to preclude iterated refinement to make a non-linear fit. Entirely satisfactory results have been found here using the single-iteration linear least-squares method, indicating that the problem is close to linear for the sizes of error encountered and that a non-linear fit is not necessary. However, if a non-linear fit was desired then the crystal parameters could be calculated by applying equation (3) after calculating the global-parameter updates. This would take relatively little time compared with the rest of the calculation, and so incorporating the Millepede principle into a non-linear algorithm may also lead to a speed benefit for those approaches.

## 8. Conclusions and outlook

Compared with spot-deviation approaches, and in common with full-matrix least-squares approaches, detector geometry refinement using Millepede takes into account the inherent mutual dependence between the detector parameters and the crystal parameters. Compared with full-matrix least-squares approaches, it involves a much smaller matrix calculation and is therefore able to refine the detector geometry with dramatically smaller computational requirements. The approach is faster even when compared with sparse matrix approaches, which already make some use of the special structure of the refinement problem.

The speed of computation makes it convenient to run as part of a standard analysis pipeline and means that the full set of experimental data can be used by the calculation rather than a small sample. Calibration of the detector can therefore be made a frequent, automated and routine part of the experimental-analysis setup instead of a special activity performed at longer intervals. Frequent refinement would also help to keep the geometry updates small and within the linear approximation used here.

Any type of geometrical distortion can be refined with this method, not only rigid motions of the detector modules. For example, pixel-array detectors in HEP may deviate from exact planarity, which can be modeled using a continuous deformation function (Bilka, 2022 ▸). In addition, not all detectors consist of two-dimensional pixel arrays. Other types of detector include ionization chambers where signals are detected by wires stretched across the detector volume, and the sagging of those wires due to gravity needs to be modeled (Blobel, 2006 ▸). In the same way, Millepede may be a useful approach to fitting lens distortions for serial electron diffraction experiments, which so far has only been achieved for rotation diffraction experiments (Brázda *et al.*, 2022 ▸).

The Millepede principle is not restricted to detector geometry alignment and can be used for any least-squares problem that follows the basic pattern of local and global parameters described in Section 2. In fact, a very similar principle has already been applied to multiple isomorphous replacement in macromolecular crystallography, to refine heavy-atom coordinates simultaneously with the magnitudes and phases of the structure factors (Bricogne, 1982 ▸). Previous methods did not take account of the mutual dependence between these parameters and suffered from poor convergence as a result (Sygusch, 1977 ▸). However, a single combined refinement of the coordinates and structure factors was computationally impractical at the time. This work pre-dates the development of Millepede in HEP by over a decade; it reaches an equivalent result to equation (6) via a different route based on implicit functions and the chain rule for differentiation, eliminating the local parameters prior to constructing the normal matrix.

A further problem in crystallography may be amenable to the Millepede principle: post-refinement of crystal parameters during merging of SX data (White, 2014 ▸; Ginn, Brewster *et al.*, 2015 ▸; Uervirojnangkoorn *et al.*, 2015 ▸). Here, the crystal orientations and lattice parameters (local parameters) are refined simultaneously with a set of estimated squared structure factors (global parameters), using (unmerged) measurements of the intensities. The refinement target function would include partiality estimation similar to previous work (White, 2014 ▸), and the crystal parameters would be refined to improve the agreement between measured intensities and those calculated using the partiality estimates and current estimates of the squared structure factors. In the end, only the ‘global’ squared structure factors are required, not the individual crystal parameters, but the calculation would directly take into account their mutual dependence. Current approaches to post-refinement separate the local and global calculations and alternate between them. Due to the large number of global parameters (reflections), it is questionable whether any speed benefit would result; however, the Millepede approach may have better convergence properties, and it could resolve questions about the uniqueness of the solution (White, Mariani *et al.*, 2016 ▸) because the eigenvectors of the normal matrix would immediately reveal weakly constrained alterations to the overall dataset.

Many other problems, as well as solutions, of this type surely exist across other scientific fields.

## 9. Software availability

The alignment procedure has been available as part of *CrystFEL* since version 0.11.0, with several improvements made in versions 0.11.1 and 0.12.0. *CrystFEL* is free and open-source software, available from https://www.desy.de/~twhite/crystfel or via https://doi.org/10.5281/zenodo.13904047. The download package includes the alignment test program described in Section 4. *Millepede-II* is available for download from https://gitlab.desy.de/millepede/millepede-ii.

## Figures and Tables

**Table 1:** Translations and rotations of panels in the simulation test, including excitation-error terms: applied perturbations (injected), corrections determined by the first and second iterations of Millepede, the total corrections from both iterations (total), and the residual offsets after correction

		Translation (mm)			Rotation (°)		
		*x*	*y*	*z*	*x*	*y*	*z*
Injected	q0	0.200	0.000	1.000	0.000	0.000	0.000
	q1	0.000	0.000	1.000	0.000	0.000	0.000
	q2	0.002	0.000	1.447	0.000	0.500	0.000
	q3	0.000	0.000	1.000	0.000	0.000	0.000

First iteration	q0	−0.187	−0.007	−1.023	−0.002	−0.012	0.005
	q1	−0.001	0.001	−1.008	−0.001	0.002	−0.001
	q2	−0.003	0.002	−1.455	0.008	−0.493	−0.003
	q3	0.000	0.001	−1.016	0.003	−0.004	0.000

Second iteration	q0	−0.014	0.008	0.025	0.002	0.013	−0.006
	q1	0.000	−0.001	0.007	0.002	−0.002	0.001
	q2	0.003	−0.003	0.012	−0.010	−0.006	0.004
	q3	0.002	0.000	0.017	−0.003	0.004	0.001

Total	q0	−0.201	0.001	−0.998	0.000	0.001	−0.001
	q1	−0.001	0.000	−1.001	0.001	0.000	0.000
	q2	0.000	−0.001	−1.443	−0.002	−0.499	0.001
	q3	0.002	0.001	−0.999	0.000	0.000	0.001

Residual offsets	q0	−0.001	0.001	0.002	0.000	0.001	−0.001
	q1	−0.001	0.000	−0.001	0.001	0.000	0.000
	q2	0.002	−0.001	0.004	−0.002	0.001	0.001
	q3	0.002	0.001	0.001	0.000	0.000	0.001

**Table 2:** Translations and rotations of panels in the simulation test, without excitation-error terms: applied perturbations (injected), corrections determined by the first and second iterations of Millepede, the total corrections from both iterations (total), and the residual offsets after correction

		Translation (mm)			Rotation (°)		
		*x*	*y*	*z*	*x*	*y*	*z*
Injected	q0	0.200	0.000	1.000	0.000	0.000	0.000
	q1	0.000	0.000	1.000	0.000	0.000	0.000
	q2	0.002	0.000	1.447	0.000	0.500	0.000
	q3	0.000	0.000	1.000	0.000	0.000	0.000

First iteration	q0	−0.184	−0.007	−1.034	0.001	−0.014	0.003
	q1	0.002	0.001	−0.999	−0.001	−0.007	−0.004
	q2	0.000	0.001	−1.440	0.003	−0.505	−0.001
	q3	0.006	−0.006	−1.009	−0.003	−0.006	0.003

Second iteration	q0	−0.015	0.010	0.040	0.003	0.014	−0.005
	q1	0.000	−0.001	0.010	0.004	0.002	0.005
	q2	0.001	−0.001	0.006	−0.005	0.000	0.003
	q3	−0.004	0.005	0.019	0.001	0.006	−0.003

Total	q0	−0.199	0.003	−0.994	0.004	0.000	−0.002
	q1	0.002	0.000	−0.989	0.003	−0.005	0.001
	q2	0.001	0.000	−1.434	−0.002	−0.505	0.002
	q3	0.002	−0.001	−0.990	−0.002	0.000	0.000

Residual offsets	q0	0.001	0.003	0.006	0.004	0.000	−0.002
	q1	0.002	0.000	0.011	0.003	−0.005	0.001
	q2	0.003	0.000	0.013	−0.002	−0.005	0.002
	q3	0.002	−0.001	0.010	−0.002	0.000	0.000

**Table 3:** Total panel movements resulting from three rounds of refinement on experimental data from 5-HT_2B_ serotonin receptor crystals

	Translation (mm)			Rotation (°)		
	*x*	*y*	*z*	*x*	*y*	*z*
q0a0	−0.455	−0.564	−2.136	−0.468	−0.091	−0.133
q0a1	−0.404	−0.565	−2.313	−0.468	−0.091	−0.133
q0a2	−0.455	−0.508	−2.172	−0.468	−0.091	−0.133
q0a3	−0.404	−0.510	−2.348	−0.468	−0.091	−0.133
q0a4	−0.352	−0.674	−2.417	−0.468	−0.091	−0.133
q0a5	−0.352	−0.623	−2.453	−0.468	−0.091	−0.133
q0a6	−0.406	−0.673	−2.228	−0.468	−0.091	−0.133
q0a7	−0.406	−0.623	−2.263	−0.468	−0.091	−0.133
q0a8	−0.242	−0.572	−2.867	−0.468	−0.091	−0.133
q0a9	−0.293	−0.572	−2.691	−0.468	−0.091	−0.133
q0a10	−0.242	−0.627	−2.830	−0.468	−0.091	−0.133
q0a11	−0.293	−0.626	−2.653	−0.468	−0.091	−0.133
q0a12	−0.247	−0.565	−2.854	−0.468	−0.091	−0.133
q0a13	−0.247	−0.514	−2.888	−0.468	−0.091	−0.133
q0a14	−0.302	−0.564	−2.663	−0.468	−0.091	−0.133
q0a15	−0.302	−0.514	−2.697	−0.468	−0.091	−0.133

q1a0	−0.033	−0.492	−1.705	0.737	−0.381	0.113
q1a1	−0.031	−0.536	−1.852	0.737	−0.381	0.113
q1a2	0.017	−0.490	−2.006	0.737	−0.381	0.113
q1a3	0.018	−0.534	−2.151	0.737	−0.381	0.113
q1a4	−0.127	−0.584	−1.404	0.737	−0.381	0.113
q1a5	−0.083	−0.583	−1.683	0.737	−0.381	0.113
q1a6	−0.128	−0.537	−1.249	0.737	−0.381	0.113
q1a7	−0.083	−0.536	−1.527	0.737	−0.381	0.113
q1a8	−0.036	−0.674	−2.276	0.737	−0.381	0.113
q1a9	−0.035	−0.629	−2.132	0.737	−0.381	0.113
q1a10	−0.083	−0.676	−1.977	0.737	−0.381	0.113
q1a11	−0.083	−0.631	−1.833	0.737	−0.381	0.113
q1a12	−0.027	−0.669	−2.304	0.737	−0.381	0.113
q1a13	0.017	−0.667	−2.582	0.737	−0.381	0.113
q1a14	−0.029	−0.622	−2.147	0.737	−0.381	0.113
q1a15	0.017	−0.620	−2.424	0.737	−0.381	0.113

q2a0	0.152	−0.041	−1.768	−0.202	0.719	−0.097
q2a1	0.115	−0.041	−1.693	−0.202	0.719	−0.097
q2a2	0.150	−0.083	−2.064	−0.202	0.719	−0.097
q2a3	0.114	−0.083	−1.987	−0.202	0.719	−0.097
q2a4	0.081	0.038	−1.035	−0.202	0.719	−0.097
q2a5	0.080	0.001	−1.307	−0.202	0.719	−0.097
q2a6	0.121	0.038	−1.117	−0.202	0.719	−0.097
q2a7	0.120	0.001	−1.389	−0.202	0.719	−0.097
q2a8	0.001	−0.037	−1.419	−0.202	0.719	−0.097
q2a9	0.037	−0.038	−1.497	−0.202	0.719	−0.097
q2a10	0.002	0.003	−1.127	−0.202	0.719	−0.097
q2a11	0.039	0.003	−1.203	−0.202	0.719	−0.097
q2a12	0.004	−0.043	−1.470	−0.202	0.719	−0.097
q2a13	0.002	−0.080	−1.741	−0.202	0.719	−0.097
q2a14	0.043	−0.043	−1.550	−0.202	0.719	−0.097
q2a15	0.042	−0.080	−1.822	−0.202	0.719	−0.097

q3a0	−0.225	0.027	−1.445	−0.567	0.627	0.117
q3a1	−0.226	0.070	−1.684	−0.567	0.627	0.117
q3a2	−0.273	0.025	−1.676	−0.567	0.627	0.117
q3a3	−0.274	0.068	−1.914	−0.567	0.627	0.117
q3a4	−0.132	0.115	−1.467	−0.567	0.627	0.117
q3a5	−0.176	0.115	−1.682	−0.567	0.627	0.117
q3a6	−0.130	0.071	−1.212	−0.567	0.627	0.117
q3a7	−0.174	0.070	−1.427	−0.567	0.627	0.117
q3a8	−0.224	0.203	−2.409	−0.567	0.627	0.117
q3a9	−0.223	0.161	−2.173	−0.567	0.627	0.117
q3a10	−0.176	0.205	−2.178	−0.567	0.627	0.117
q3a11	−0.175	0.162	−1.941	−0.567	0.627	0.117
q3a12	−0.231	0.200	−2.418	−0.567	0.627	0.117
q3a13	−0.276	0.199	−2.631	−0.567	0.627	0.117
q3a14	−0.230	0.154	−2.160	−0.567	0.627	0.117
q3a15	−0.275	0.153	−2.374	−0.567	0.627	0.117

**Table 4:** Panel movements from a fourth round of refinement on experimental data from 5-HT_2B_

	Translation (mm)			Rotation (°)		
	*x*	*y*	*z*	*x*	*y*	*z*
q0a0	0.000	−0.006	−0.005	−0.003	−0.001	−0.005
q0a1	0.002	−0.006	−0.006	−0.003	−0.001	−0.005
q0a2	0.000	−0.004	−0.005	−0.003	−0.001	−0.005
q0a3	0.002	−0.004	−0.007	−0.003	−0.001	−0.005
q0a4	0.004	−0.011	−0.006	−0.003	−0.001	−0.005
q0a5	0.004	−0.009	−0.007	−0.003	−0.001	−0.005
q0a6	0.002	−0.011	−0.005	−0.003	−0.001	−0.005
q0a7	0.002	−0.009	−0.006	−0.003	−0.001	−0.005
q0a8	0.009	−0.007	−0.010	−0.003	−0.001	−0.005
q0a9	0.007	−0.007	−0.009	−0.003	−0.001	−0.005
q0a10	0.009	−0.009	−0.009	−0.003	−0.001	−0.005
q0a11	0.006	−0.009	−0.008	−0.003	−0.001	−0.005
q0a12	0.008	−0.006	−0.010	−0.003	−0.001	−0.005
q0a13	0.008	−0.004	−0.010	−0.003	−0.001	−0.005
q0a14	0.006	−0.006	−0.008	−0.003	−0.001	−0.005
q0a15	0.006	−0.004	−0.009	−0.003	−0.001	−0.005

q1a0	0.005	−0.006	−0.005	0.064	−0.024	−0.001
q1a1	0.005	−0.005	−0.015	0.064	−0.024	−0.001
q1a2	0.005	−0.006	−0.032	0.064	−0.024	−0.001
q1a3	0.005	−0.005	−0.041	0.064	−0.024	−0.001
q1a4	0.006	−0.006	0.028	0.064	−0.024	−0.001
q1a5	0.006	−0.005	0.004	0.064	−0.024	−0.001
q1a6	0.006	−0.006	0.038	0.064	−0.024	−0.001
q1a7	0.006	−0.006	0.013	0.064	−0.024	−0.001
q1a8	0.006	−0.004	−0.040	0.064	−0.024	−0.001
q1a9	0.006	−0.004	−0.031	0.064	−0.024	−0.001
q1a10	0.006	−0.004	−0.014	0.064	−0.024	−0.001
q1a11	0.006	−0.004	−0.005	0.064	−0.024	−0.001
q1a12	0.006	−0.004	−0.043	0.064	−0.024	−0.001
q1a13	0.005	−0.003	−0.067	0.064	−0.024	−0.001
q1a14	0.006	−0.004	−0.033	0.064	−0.024	−0.001
q1a15	0.005	−0.004	−0.057	0.064	−0.024	−0.001

q2a0	0.000	0.004	−0.014	−0.015	0.025	0.002
q2a1	0.001	0.004	−0.008	−0.015	0.025	0.002
q2a2	0.000	0.004	−0.024	−0.015	0.025	0.002
q2a3	0.001	0.004	−0.018	−0.015	0.025	0.002
q2a4	0.002	0.002	0.018	−0.015	0.025	0.002
q2a5	0.002	0.003	0.009	−0.015	0.025	0.002
q2a6	0.001	0.002	0.011	−0.015	0.025	0.002
q2a7	0.001	0.003	0.002	−0.015	0.025	0.002
q2a8	0.003	0.004	0.012	−0.015	0.025	0.002
q2a9	0.003	0.004	0.006	−0.015	0.025	0.002
q2a10	0.003	0.003	0.022	−0.015	0.025	0.002
q2a11	0.003	0.003	0.016	−0.015	0.025	0.002
q2a12	0.003	0.004	0.010	−0.015	0.025	0.002
q2a13	0.003	0.004	0.000	−0.015	0.025	0.002
q2a14	0.002	0.004	0.003	−0.015	0.025	0.002
q2a15	0.002	0.004	−0.006	−0.015	0.025	0.002

q3a0	−0.004	0.003	0.091	−0.292	−0.019	0.005
q3a1	−0.004	0.004	0.097	−0.292	−0.019	0.005
q3a2	−0.006	0.002	−0.029	−0.292	−0.019	0.005
q3a3	−0.006	0.002	−0.022	−0.292	−0.019	0.005
q3a4	−0.001	0.007	0.340	−0.292	−0.019	0.005
q3a5	−0.002	0.006	0.230	−0.292	−0.019	0.005
q3a6	−0.001	0.007	0.332	−0.292	−0.019	0.005
q3a7	−0.002	0.005	0.222	−0.292	−0.019	0.005
q3a8	−0.004	0.006	0.133	−0.292	−0.019	0.005
q3a9	−0.004	0.005	0.125	−0.292	−0.019	0.005
q3a10	−0.002	0.007	0.251	−0.292	−0.019	0.005
q3a11	−0.002	0.007	0.244	−0.292	−0.019	0.005
q3a12	−0.004	0.006	0.115	−0.292	−0.019	0.005
q3a13	−0.006	0.004	0.005	−0.292	−0.019	0.005
q3a14	−0.004	0.005	0.108	−0.292	−0.019	0.005
q3a15	−0.006	0.004	−0.002	−0.292	−0.019	0.005

**Table 5:** Time taken to refine a detector model as described in Section 4, for a wide range of dataset sizes

Number of patterns	Time taken (s)
100	0.162
999	0.803
5000	3.47
9999	6.72
49 992	37.2
100 980	78.2

## Footnotes

1 This speed measurement was performed using the data described in Section 5, with seven parallel processes on a desktop computer equipped with a four-core (eight thread) Intel Xeon W-2225 processor at 4.1 GHz clock frequency and 16 GB of memory.
